# Proteomic Analysis of the Reproductive Organs of the Hermaphroditic Gastropod *Lymnaea stagnalis* Exposed to Different Endocrine Disrupting Chemicals

**DOI:** 10.1371/journal.pone.0081086

**Published:** 2013-11-19

**Authors:** Arnaud Giusti, Pierre Leprince, Gabriel Mazzucchelli, Jean-Pierre Thomé, Laurent Lagadic, Virginie Ducrot, Célia Joaquim-Justo

**Affiliations:** 1 Laboratory of Animal Ecology and Ecotoxicology, Centre of Analytical Research and Technology (CART), Liege University, Liège, Belgium; 2 INRA (Institut National de la Recherche Agronomique), UMR0985 Ecologie et Santé des Ecosystèmes, Equipe Ecotoxicologie et Qualité des Milieux Aquatiques, Rennes, France; 3 GIGA-Neuroscience, Liège University, Liège, Belgium; 4 Laboratory of Mass Spectrometry, GIGA-R, Liège University, Liège, Belgium; Gettysburg College, United States of America

## Abstract

Many studies have reported perturbations of mollusc reproduction following exposure to low concentrations (ng/L range) of endocrine disrupting chemicals (EDCs). However, the mechanisms of action of these molecules on molluscs are still poorly understood. Investigation of the modifications of protein expression in organisms exposed to chemicals using proteomic methods can provide a broader and more comprehensive understanding of adverse impacts of pollution on organisms than conventional biochemical biomarkers (e.g., heat-shock proteins, metallothioneins, GST, EROD). In this study we have investigated the impacts of four chemicals, which exhibit different endocrine disrupting properties in vertebrates, on the proteome of the hermaphroditic freshwater pulmonate gastropod *Lymnaea stagnalis* after 21 days of exposure. Testosterone, tributyltin, chlordecone and cyproterone acetate were chosen as tested compounds as they can induce adverse effects on the reproduction of this snail. The 2D-DIGE method was used to identify proteins whose expression was affected by these compounds. In addition to modifying the expression of proteins involved in the structure and function of the cytoskeleton, chemicals had impacts on the expression of proteins involved in the reproduction of *L. stagnalis*. Exposure to 19.2 µg/L of chlordecone increased the abundance of ovipostatin, a peptide transmitted during mating through seminal fluid, which reduces oviposition in this species. The expression of yolk ferritin, the vitellogenin equivalent in *L. stagnalis*, was reduced after exposure to 94.2 ng Sn/L of tributyltin. The identification of yolk ferritin and the modification of its expression in snails exposed to chemicals were refined using western blot analysis. Our results showed that the tested compounds influenced the abundance of yolk ferritin in the reproductive organs. Alteration in proteins involved in reproductive pathways (e.g., ovipostatin and yolk ferritin) could constitute relevant evidence of interaction of EDCs with reproductive pathways that are under the control of the endocrine system of *L. stagnalis*.

## Introduction

An Endocrine Disrupting Chemical (EDC) is defined as an exogenous substance or mixture that alters function(s) of the endocrine system and consequently causes adverse health effects in an intact organism, or its progeny, or (sub) populations [1]. A wide variety of chemicals are known to be endocrine disruptors: natural and synthetic hormones, pesticides, industrial by-products and other chemicals produced in the plastic industry [1]. Mechanisms of action of EDCs can be mediated through receptor binding, inhibition of hormone synthesis, metabolism and transport [1,2]. Impacts of EDCs on wildlife are numerous and differ highly between taxa, species and even between reproductive statuses of organisms.

Many compounds are able to induce adverse impacts in mollusc species in the environment, e.g., superfeminisation and imposex in gastropod species [3,4], intersex in bivalves [5,6]. Furthermore, laboratory and field studies have shown that mollusc reproduction is altered by exposure to low concentrations (ng/L range) of these molecules, indicating that mollusc species are particularly sensitive to EDCs. Therefore, some mollusc species are already used as bio-indicators in the field (e.g. *Nucella lapillus*, *Littorina littorea*) [7,8] or as test species in laboratory (e.g., *Potamopyrgus antipodarum*, *Marisa cornuarietis*) [9,10]. The hermaphroditic gastropod *Lymnaea stagnalis* has been shown to be responsive to EDCs such as organotin compounds (i.e., TBT and TPT), some pesticides and industrial products (e.g., vinclozolin, nonylphenol) [11-14]. Even though our knowledge of mollusc endocrinology is rather limited, the neurohormonal control of reproduction is reasonably well understood in *L. stagnalis* [15-17]. Moreover, genomic resources have recently been improved by pyrosequencing of cDNAs from *L. stagnalis*, expanding the databases for protein identification in genomic and proteomic approaches for this species [18]. Finally, this species has been proposed as a candidate species for the development of guidelines for the testing and the assessment of endocrine disruptors in molluscs [19].

Proteomic analyses in ecotoxicology aim to obtain a comprehensive and quantitative description of protein expression and modification (e.g., abundance, activity, structure, post-translational modification) following exposure to environmental stress conditions [20]. Temperature fluctuation, parasitism, and exposure to environmental pollutants lead to alterations of the protein expression in stressed animals [21,22]. Investigations of the modification of protein expression in organisms exposed to chemicals using proteomic methods can provide a broader and more comprehensive understanding of adverse impacts of pollution on the organisms than conventional biochemical biomarkers (e.g., heat-shock proteins, metallothioneins, GST, EROD) [23]. As proteomic is a large-scale, hypotheses-free approach, knowledge on the exact mechanisms of action of the chemicals is not required [23-27]. Therefore analysis of the impacts of one or more environmental contaminants on the proteome of a species can provide insights on the mechanisms of action of molecules and pinpoint a number of potential biomarkers of exposure to specific pollutants [22,26,28]. Even though few studies have investigated the impacts of chemicals on mollusc using proteomic methods, specific biomarkers and protein expression signatures were observed following exposure to particular toxicants [24,27,29,30]. However, the majority of these studies were conducted on bivalve species and the impacts of endocrine disrupting chemicals on protein expression have rarely been reported in gastropods [31].

In this study we have investigated the impacts of four chemicals (i.e., testosterone, cyproterone acetate, tributyltin, and chlordecone), which exhibit different endocrine disrupting properties in vertebrates, on the proteome of *L. stagnalis* after 21 days of exposure. Chemicals were chosen on the basis of their affinities to the vertebrate oestrogen and androgen receptors, as assessed by *in vitro* screening tests. Testosterone is the natural ligand of the androgen receptor in vertebrates and endogenous concentrations of this steroid hormone were found in *L. stagnalis* [32] as well as in other molluscs [33,34]. Laboratory studies have reported that testosterone endogenous concentrations in mollusc tissues vary according to the reproductive status [35,36]. In laboratory experiments, exposure of molluscs to testosterone led to alterations of oocytes in female bivalves [37] and potentiated spawning in males [38,39]. These observations suggest that testosterone likely play physiological roles in mollusc reproduction. The synthetic steroid pharmaceutical cyproterone acetate acts as an anti-androgen by competitively blocking androgen receptors in vertebrates [40]. Moreover, cyproterone acetate decreased penis length and spermatogenesis in the gastropods, *Hinia reticulata* and *N*. *lapillus* [10]. Chlordecone binds to the vertebrate oestrogen receptor [41] albeit with relatively low affinity [42]. The impact of chlordecone on mollusc reproduction was never assessed. Finally, TBT is the only proven endocrine disruptor in molluscs (i.e., imposex induction has been reported in more than 200 species [43]), however mechanisms of imposex induction by TBT are still unclear [44]. Other modes of action, such as regulation of aromatase activity, has been demonstrated or suggested for the tested chemicals in mammals [45-47]. Therefore, due to their different mechanisms of actions known in vertebrates, these molecules might differently affect protein expression in reproductive organs of the hermaphroditic freshwater gastropod *L. stagnalis*. This study investigates the impacts of 21-day exposures to the four putative endocrine disruptors on the proteome of the reproductive organs of *L. stagnalis*. The two-dimensional differential fluorescence in gel electrophoresis method (2D-DIGE) was used to identify proteins significantly altered by the exposures. Western Blot analysis was further used to analyse the alteration of the expression of a protein involved in *L. stagnalis* reproduction, the yolk ferritin. The impacts of the chemicals on protein expression and the potential reproductive impacts linked to alteration of these proteins were discussed. 

## Materials and Methods

### 1: Animals and Exposure Experiment

*L. stagnalis* (Linnaeus, 1758) (Mollusca, Gastropoda, Pulmonata, Heterobranchia), RENILYS^®^ strain, has been reared at the INRA Experimental Unit of Aquatic Ecology and Ecotoxicology (Rennes, France) under laboratory conditions as previously described [48]. Young adults of homogenous size (22.5 ± 2.5 mm) and age (4 ± 0.5 months) were sampled from the culture and acclimatised to test conditions for 48 hours prior to chemical exposure. 

Analytical standards of testosterone (≥98% purity, CAS Nr 58-22-0), cyproterone acetate (≥98% purity, CAS Nr 427-51-0), tributyltin hydride (≥97% purity, CAS Number 688-73-3) and chlordecone (Pestanal^®^ grade, CAS Nr 143-50-0) were purchased from Sigma-Aldrich (Schnelldorf, Germany). Exposure media consisted in dechlorinated, charcoal-filtered tap water (i.e., similar to culture medium; pH = 7.7 ± 0.2, conductivity = 623 ± 60 µS/cm, dissolved oxygen = 7.3 ± 2 mg/L, water hardness = 254 ± 7 mg CaCo_3_/L) contaminated with stock solutions prepared in analytical grade acetone (99.9 % purity). Concentrations of stock solutions were chosen to ensure addition of the same amount of solvent among treatments (i.e., 100 µL/L, except for the water control) as recommended by OECD [49]. Six replicates (each with five snails in 1 L glass beaker) of each chemical concentration (Table 1), water controls and solvent controls were randomly distributed in the exposure room. Tests were conducted at constant temperature (20.5 ± 0.6°C) and photoperiod (14:10 Light:Dark) as previously described [12]. Snails were fed every other day with 2.5g of rinsed organic lettuce per exposure beaker. Exposure media, as well as water controls and solvent controls, were renewed every 2 days to ensure maintaining exposure concentration and physico-chemical properties of test water during the experiment duration. After 21 days of exposure, snails were frozen at -80°c until proteomic analysis. 

**Table 1:** Nominal and time-weighted average exposure concentrations (AEC) of chemicals in water.

Chemicals	Nominal concentration	Average exposure concentration (AEC)
Water controls	-	-
Solvent Controls	-	-
Testosterone (T)	2 ng/L	0.3 ng/L
	10 ng/L	1.4 ng/L
	50 ng/L	6.8 ng/L
Cyproterone acetate (CPA)	2 µg/L	1.1 µg/L
	50 µg/L	28.7 µg/L
Chlordecone (CLD)	4,5 µg/L	2.1 µg/L
	50 µg/L	19.6 µg/L
Tributyltin (TBT)	45 ng Sn/L	19.2 ng Sn/L
	220 ng Sn/L	94.2 ng Sn/L

### 2: Chemical Analysis

Exposure water was sampled at the beginning, mid-term and end of the experiment. Three replicates (200 mL) were taken per concentration. Each replicate consists in a pool of 100 mL of water sampled from two beakers. Samples were taken 15 minutes and 48 hours after water renewal and stored in glass flasks at -20°C until analysis. Testosterone and cyproterone acetate were extracted using C-18 solid phase extraction (SPE) and determined by high-resolution liquid chromatography – mass spectrometry (HPLC-MS-MS). Chlordecone was extracted by manually handshaking 5 mL of water with a volume of 5 mL of dichloromethane. Extraction of tributyltin was performed as described in the ISO method for organotin analysis in water [50]. Analysis and quantification of chlordecone and tributyltin concentrations were performed by capillary gas chromatography - mass spectrometry (GC-MS-MS). Actual exposure concentrations were calculated as the time-weighted average of measured concentrations (AEC) over the test period [51] and were further used instead of nominal concentrations (Table 1). 

### 3. 2D-DIGE (Two-Dimensional Differential In-Gel Electrophoresis)

#### 3.1. Protein Extraction

After 21 days of exposure, four of the six replicates were randomly chosen per experimental condition. One snail was randomly sampled in each of these four replicates and frozen at -80°c until protein extraction. Reproductive tissues were isolated by dissection of frozen individuals and weighted. Protein extraction was performed as described in previous studies conducted on arthropods and bivalves [52-54]. Samples were crushed in a volume of lysis buffer / ASB14 (7 M urea, 2 M thiourea, 30 mM Tris pH 8.5 buffer including 2% ASB14) equivalent to ten times the fresh weight of the sample. A hundred microlitres of homogenate were taken and submitted to subsequent extraction steps. Samples were sonicated for 10 min, incubated 15 min with 1 µL benzonase nuclease (≥250 units/µL, Sigma-Aldrich, Schnelldorf, Germany) and centrifuged at 20000 g at 4°c for 30 min. Supernatants were collected and proteins were precipitated using a 2D Clean-Up Kit according to the manufacturer’s instructions (GE Healthcare, Diegem, Belgium). Protein pellets were suspended in 100 µL of lysis buffer and pH was adjusted to 8.5 with NaOH. Protein quantification was carried out using the RC DC Protein assay kit (Bio-Rad, Nazareth, Belgium). Samples were stored at -80°C until subsequent steps.

#### 3.2. CyDye Labelling

Two-dimensional Differential In-Gel separation method uses fluorescent cyanine dyes, i.e., CyDyes, as protein staining prior to protein separation. Twenty-five micrograms of proteins were labelled with 200 pmol of either Cy3 or Cy5 (GE Healthcare, Diegem, Belgium); per experimental condition, two replicates were labelled with Cy3 and two replicates with Cy5. Internal standard was obtained by pooling equal amounts of proteins (25 µg) of each biological sample and labelled with Cy2 (GE Healthcare, Diegem, Belgium). Following 30 min of incubation in darkness, the labelled samples were quenched with the addition of 0.2 µL of 10 mM Lysine (Sigma-Aldrich, Schnelldorf, Germany) and submitted to another 10 min incubation in darkness.

#### 3.3. Protein Separation

Pairs of randomly chosen Cy3 and Cy5 samples were mixed and pooled with 25 µg of Cy2-labelled internal standard. After the addition of 1% DTT (1M) and 2% of IPG Buffer 3-11 NL (GE Healthcare, Diegem, Belgium), the volume was adjusted to 450 µL with rehydration buffer 3-11 NL ASB14 (7 M urea, 2 M thiourea, 2% w/v ASB14, 25 mM DTT, and 0.6% v/v pH 3–11 NL IPG buffer). Protein focussing was performed at 20 °C on a 24 cm strip (pH 3-11 NL, GE Healthcare, Diegem, Belgium) for 23 h 45 min using an Ettan IPGphor II isoelectric focussing System (GE Healthcare, Diegem, Belgium) at 500 V for 1 h, followed by a gradient from 500 to 1000 V in 3 h. During the next 3 hours the voltage increased from 1000 V to 8000 V and the voltage was held at 8000 V to achieve 85000Vh. 

Focussed strips were equilibrated for 15 min in equilibration buffer (50 mM Tris-HCl pH 8.8, 6 M urea, 30% glycerol (v/v), 1.6% SDS (w/v)) with 1% DTT and a second equilibration step was performed for another 15 min in equilibration buffer with 5 % of iodoacetamide. For the second dimension of electrophoresis, strips were loaded on a 12.5% (w/v) polyacrylamide gel and migration was carried out at 30°C at 1W/gel and 80V for 1 hour followed by 2W/gel and 50V overnight until completion of migration using an Ettan Dalt-6 system (GE Healthcare, Diegem, Belgium). 

#### 3.4. Image Acquisition and Analysis

Gels were scanned using a Typhoon 9400 Laser scanner (GE Healthcare, Piscataway, NJ, United States) with excitation and emission wavelengths specific to each CyDye (Cy2: 488/520; Cy3: 523/580 and Cy5: 633/670 nm). 2D-Gel images were analysed using Decyder 2D Differential analysis software (v.7.0, GE Healthcare, Piscataway, NJ, United States). Differential In-Gel Analysis (DIA) module was used for spot detection and abundance measurement for each sample. Internal control was used to match the acquired gels. Protein spots intensities in samples are normalized by dividing them by the intensity of the corresponding spot on the internal standard image [55]. The differentially expressed proteins in treatments compared to water or solvent controls were detected using Biological Variance Analysis (BVA) module. The unpaired student’s t-test was used and a p-value <0.05 was considered statistically significant.

#### 3.5. Protein Identification

Protein spots that showed a significant fold change of at least 1.5 with a p<0.05 (unpaired Student’s *t*-test) in reproductive organs of *L. stagnalis* exposed to chemicals were submitted to identification. Two preparative gels were loaded with 250 µg of unlabelled proteins from either water or solvent controls and 25 µg of the internal standard. Gels were run under the same conditions as analytical gels. Spots of interest were excised using an Ettan Spotpicker robot (GE Healthcare, Piscataway, NJ, United States) and transferred to a 96-Wells plate (Eppendorf, Hamburg, Germany). Proteins were digested with 20 ng/L of trypsin (Roche, porcine, proteomic grade) for 4 h at 37°C using a Janus Robot (Perkin Elmer, Waltham, MA, USA) [56]. Resulting peptides were extracted and rehydrated in 10 µL of formic acid (1%). Automated spectra acquisition was performed using an Ultraflex II MALDI mass Spectrometer (Bruker Daltonics, Billerica, MA) under control of Flex Control v.3.0. and Flex Analysis v.3.0. software (Bruker Daltonics, Billerica, MA) in MS mode for peptide mass fingerprint (PMF, spectra acquisition mass range: 70-4000 *m/z*) followed by the MS-MS mode for peptide sequencing. Peptides identification was managed using Biotools v.3.1. software (Bruker Daltonics, Billerica, MA) with an in-house hosted Mascot v2.2.2. server. Metazoa taxonomy was used for database search with the following parameters: peptide mass tolerance of 100 ppm precision, charge state of 1+ and a maximum number of missed cleavages of 1. Carbamidomethylation of cysteines was used as a fixed modification and oxidation of methionine as a variable modification. Identification was significant for peptide mass fingerprint with a p<0.05 and a Mascott protein score ≥75. 

Further protein identification was performed using genomic resources obtained by pyrosequencing *L. stagnalis* individuals from our laboratory cultures [18]. Open reading frames (ORFs) were searched among all the snail contigs available (Great Pond Snail Contig Browser: http://genotoul-contigbrowser.toulouse.inra.fr:9095/Lymnaea_stagnalis/index.html) with the EMBOSS getorf free open source software (http://emboss.open-bio.org/wiki/Appdocs). Translation of regions of minimum 30 nucleotides was performed between methionine (start codon) and stop codons. ORFs were also searched in the reverse sequence. The ORFs obtained in Pearson FASTA format were included in our in-house Mascott library and protein identifications were performed using the previously described parameters. Significantly identified contigs were searched in NCBI database for sequence homology with a cut-off e-value of 1^-5^ as previously described [18] and available on the Great Pond Snail Contig Browser: http://genotoul-contigbrowser.toulouse.inra.fr:9095/Lymnaea_stagnalis/index.html.

### 4: Western Blot

#### 4.1. Antibody Design and Production

For antibody production, a peptide was selected in the *L. stagnalis* yolk ferritin protein sequence (Uniprot accession number: P42578) based on the hydrophobicity, antigen index and on secondary structure using dedicated software (Eurogentec, Liege, Belgium). The peptide selected was searched in the NCBI database to avoid cross detection of other proteins. The synthetic peptide (H2N-LRSFEQGSGNNYKLGC-CONH2) was coupled with ovalbumin carrier protein and was injected in rabbit. Total rabbit serum was recovered after 28 days (Eurogentec, Liege, Belgium).

#### 4.2. Protein Extraction and Separation

Four of the six replicates were randomly chosen per experimental condition. One snail was randomly sampled in each of these four replicates and frozen at -80°c until protein extraction. Reproductive organs were isolated by dissection of frozen individuals. The prostate gland was removed and samples were weighted. Proteins were extracted by homogenisation in a volume of lysis buffer (20 mM Tris pH 8.5, 150 mM NaCl, 2 mM EDTA and 0.1% Triton-X with a cocktail of protease inhibitor (Roche, Meylan, France)) equivalent to ten times the fresh weight of the sample. A hundred microlitres of homogenate were taken and submitted to subsequent extraction steps. Samples were sonicated for 30 min at room temperature and were centrifuged at 20000 g at 4°c for 30 min. Total proteins concentrations were measured using Pierce 660 nm protein assay (Fisher Scientific Inc., Rockford, IL).

A constant amount of protein (25 µg) and 5 µL of a pre-stained molecular weight ladder (PageRuler Plus Prestained Protein Ladder 10-250kDa, Fisher Scientific Inc., Rockford, IL) were loaded on a 12% polyacrylamide gel. Gels were run with electrode buffer (25 mM Tris pH 8.8, 192 mM glycine, 2% SDS (w/v)) using a Bio-Rad Mini Protean system (Bio-Rad, Hercules, CA, USA) for 1 h at 150V. Proteins were transferred on nitrocellulose membranes (Hybond ECL, Amersham/GE Healthcare, Diegem, Belgium) at 2 mA/cm^2^ during 1 h using a Trans-Blot SD Semi-Dry Transfer Cell (Bio-Rad, Hercules, CA, USA). Total proteins were stained with Ponceau red (0.2%) in 5% acetic acid and membranes were scanned for total proteins using an Image Scanner (Amersham Pharmacia Biotech, Orsay, France). Non-specific binding sites were blocked with 3% of powdered skim milk in TBS-T (pH 7.6) (20 mM Tris pH 7.6, 150 mM NaCl and 0.05% Tween-20). Membranes were incubated overnight at 4°c with anti-yolk ferritin antibody (Eurogentec, Liege, Belgium) (dilution of 1/10 in 3% powdered skim milk in TBS-T). After 3 washings in TBS-T, nitrocellulose membranes were incubated in darkness for 1 h at room temperature with anti-rabbit antibody conjugated with Alexa Fluor Cy2. After 3 washings in TBS-T followed by 1 washing in TBS, membranes were dried at 37°c for 1 h and were scanned using a Typhoon 9400 Laser scanner (GE Healthcare, Piscataway, NJ, United States) with excitation and emission wavelength specific to the CyDye (488/520 nm). Analyses were performed in triplicates for each biological sample. 

#### 4.3. Protein Quantification and Analysis

For yolk ferritin quantification, membranes were scanned as TIFF files (8 Bit, 300 dpi). Ponceau S reversible protein staining, usually applied as quality control of membrane transfer, was used to quantify total amount of protein loaded per condition by scanning membranes prior to antibody incubation. In comparison to the quantification using the “housekeeping” proteins method, which generally uses ß-actin and/or glyceraldehyde 3-phosphate dehydrogenase (GAPDH) amongst other proteins whose level is thought to be stable between conditions, Ponceau staining normalisation avoid the possibility that these housekeeping proteins abundance would also be altered by the experimental conditions [57,58]. 

Total proteins and yolk ferritin bands were quantified using the 1D gel analysis of ImageQuantTL software (GE Healthcare, Piscataway, NJ, United States). The inter-gel normalisation of the results was performed using a positive control (i.e., proteins extracted from eggs sampled in our laboratory breeding stock) as 100% reference. Therefore the results of yolk ferritin expression in samples were expressed as percentage of the yolk ferritin expression quantified in the positive control ran in the same gel. Data were checked for normality using the d'Agostino and Pearson test [59] and for homoscedasticity using the Bartlett and Kendall test [60]. Statistical differences between water and solvent controls were analysed using a Mann-Whitney test. Differences between treatments and controls were tested using One-Way ANOVA and followed by a Dunnett’s *post-hoc* test. Statistical analyses were performed using GraphPad Prism 5.0 software (GraphPad Software, San Diego, CA, USA).

## Results

### 1. 2D: DIGE

**Figure 1:**
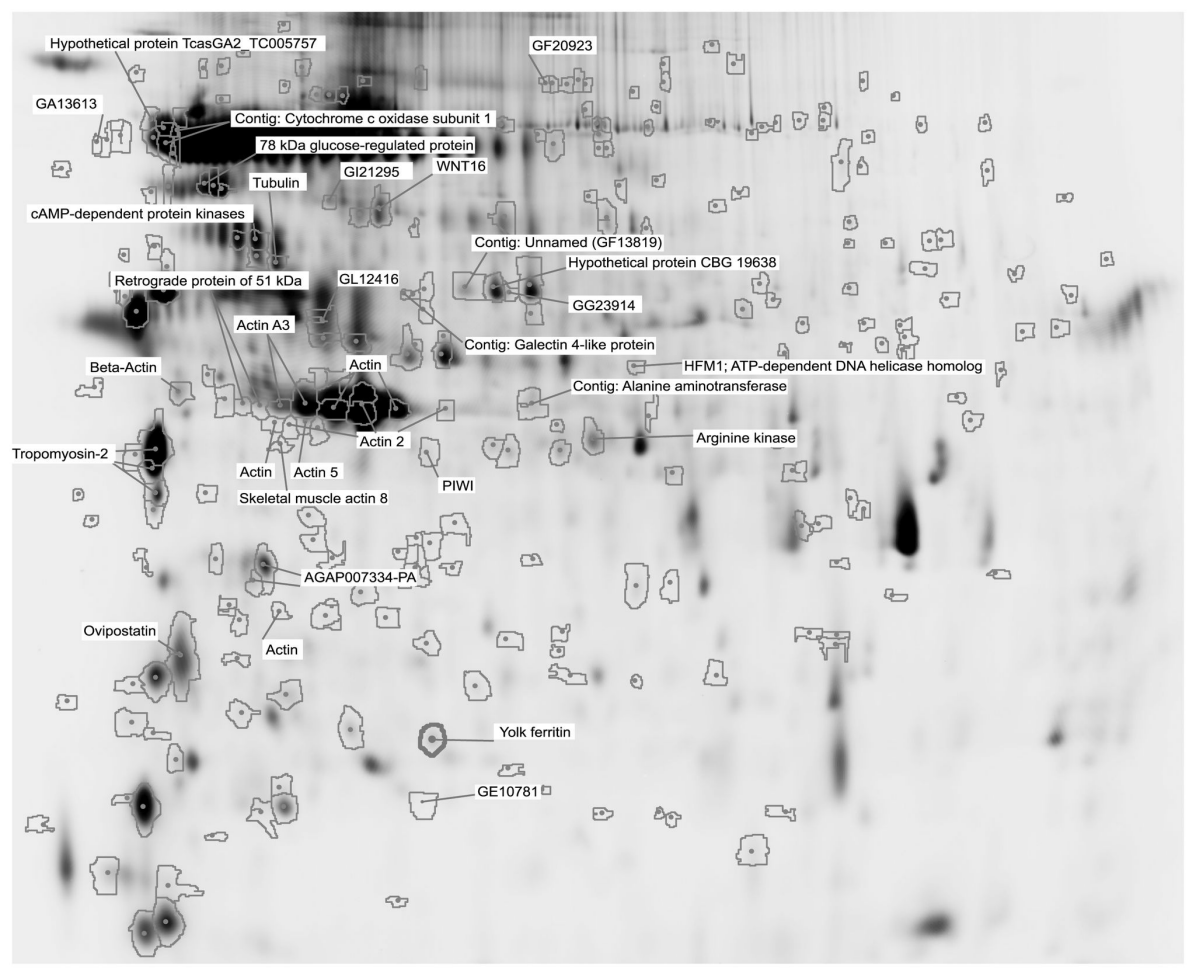
Gel master and location of the protein spots picked (contours are protein spots with at least 1.5 fold expression change, p<0.05, student *t*-test) and proteins significantly identified with the NCBI Metazoa database and with the *Lymnaea* *stagnalis* contig database.

**Figure 2:**
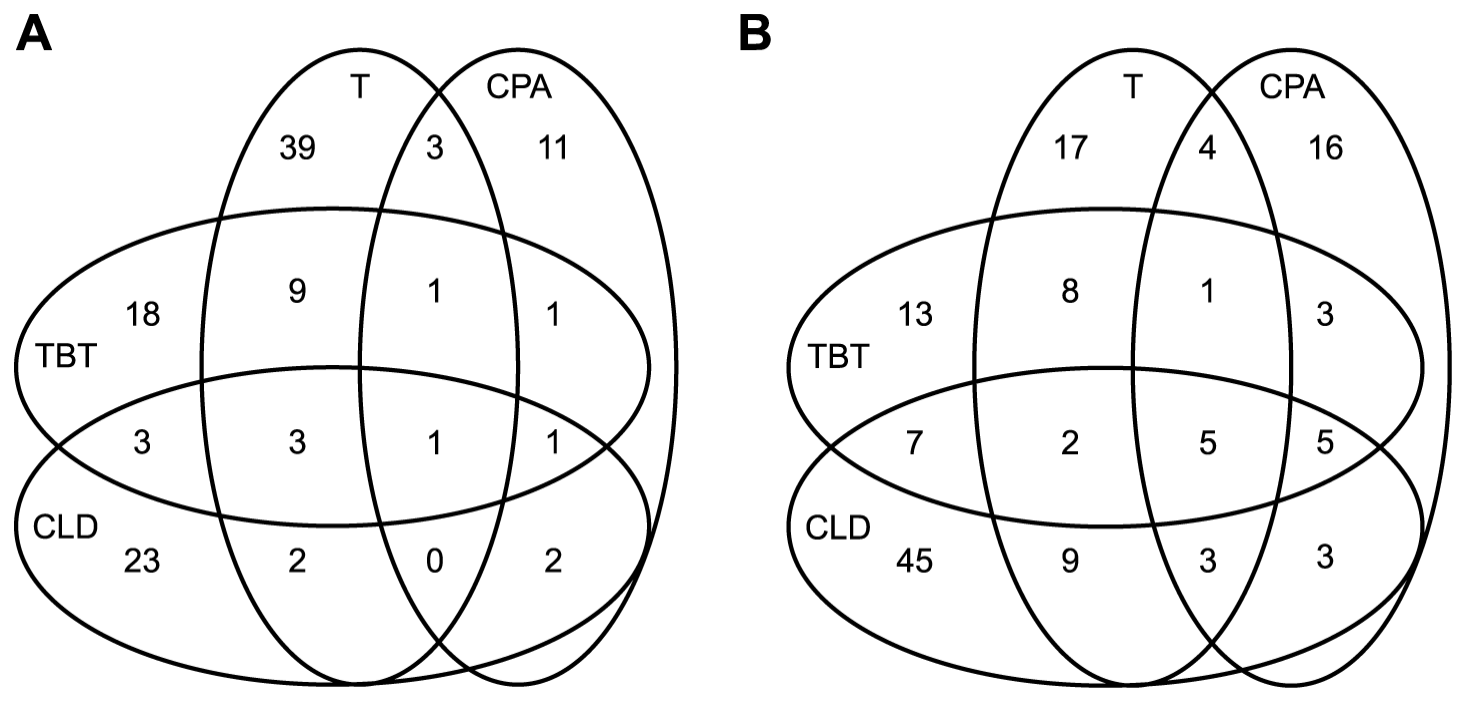
Venn diagrams of proteins with at least 1.5 fold expression change (p<0.05, student *t*-test) in reproductive organs of *Lymnaea* *stagnalis* exposed to testosterone (T), cyproterone acetate (CPA), tributyltin and chlordecone during 21 days compared to (A) water controls and (B) solvent controls.

In this study, we have investigated the impact of four chemicals, which have different endocrine disrupting properties in vertebrates, on the expression of proteins in the reproductive organs of the hermaphrodite gastropod *L. stagnalis*. From the master gel image it was possible to detect more than 4000 spots containing protein species expressed in the reproductive organs of *L. stagnalis* over the selected pH range (pH 3-11) (Figure 1). All tested chemicals had a significant impact on the expression of several proteins extracted from the reproductive organs. According to the statistical threshold (p<0.05, unpaired Student’s *t*-test) and the 1.5 fold change criterion, a total of 117 and 141 proteins were detected as differently expressed in treated samples compared to water and to solvent controls, respectively (Figure 2). Solvent alone had an impact on the expression of 18 proteins compared to water controls, amongst which 9 proteins were not altered following exposure to the tested chemicals. Proteins significantly altered in the solvent controls were mainly upregulated (13) compared to the water controls whereas after chemical exposure, the majority of proteins were downregulated when compared to water controls (Table S1). Proteins responsive to at least 2 of the tested chemicals or to different concentrations of a chemical were always altered in a same way (i.e., either up- or downregulation). The abundance of 58 protein spots was significantly altered following exposure to testosterone compared to water controls whereas tributyltin, chlordecone and cyproterone acetate had an impact on 37, 35 and 20 protein spots, respectively (Figure 2 A). Compared to the protein expression in solvent controls, chlordecone, tributyltin and cyproterone acetate had an impact on a greater number of protein spot (79, 44 and 40 spots, respectively), while exposure to testosterone altered 49 spots (Figure 2 B). Interestingly, we observed that most of the proteins altered following exposure to EDCs were specifically disrupted by a single compound as 78 and 65% of spots were only disrupted by one chemical compared to water and to solvent controls, respectively (Figure 2). Only 1 and 5 proteins were simultaneously altered by all four tested chemicals, compared to the water or the solvent controls, respectively (Table S1).

### 2: Protein Identification

Among the 144 protein spots excised, the Mascott search within the metazoa database provided 57 significant identifications, among which only 13 proteins were significantly identified by MS-MS. Forty proteins were identified as homologous to invertebrate proteins and 57 % of these proteins were significantly identified in mollusc species (Table 2). The DNA contigs obtained after pyrosequencing of the genome of *L. stagnalis* were used to generate translated open reading frames (ORFs), which were added to the Mascott library, in order to improve the protein identifications. However, only 20 ORFs showed a significant matching score and only 7 proteins were newly identified. Half of these ORFs were identified by MS-MS analyses (Table S1). 

**Table 2:** Proteins with at least 1.5-fold expression change (p<0.05, student *t*-test) in reproductive organs of *Lymnaea stagnalis* after 21 days of exposure to chemicals significantly identified with the NCBI Metazoa database and with the *Lymnaea stagnalis* contig data base.

Spot number	MW		pI		Score Mascott	MS Coverage		p-value	Protein identification	Accession number	Organism	Taxonomy (Phylum, Class)	Functions	Identification	
1411	51678		5.2		95	31		6.1^-4^	Retrograde protein of 51 kDa	gi|74912853	*Lymnaea stagnalis*	Mollusca, Gastropoda	Cytoskeleton and muscle movements	PMF	
1474	49148		4.9		103	43		9.6^e-5^	Tubulin	gi|156383445	*Nematostella vectensis*	Cnidaria, Anthozoa		PMF	
1736	136747		7.1		90	22		0.002	GL12416	gi|195157178	*Drosophila persimilis*	Arthropoda, Insecta		PMF	
1991	42081		5.3		91	41		<0.05	Actin	gi|113290	*Aplysia californica*	Mollusca, Gastropoda		MS-MS	
	8125		9.9		34	18		<0.05	Contig: Actin	GW7IPVU02F2ZX3	*Crassostrea gigas*	Mollusca, Bivalvia		MS-MS	
2067	42036		5.3		141	64		1.5^e-8^	Beta-actin	gi|110617755	*Mizuhopecten yessoensis*	Mollusca, Bivalvia		PMF	
2114	42252		5.3		132	7		<0.05	Actin A3	gi|1881572	*Biomphalaria glabrata*	Mollusca, Gastropoda		MS-MS	
	16066		6.6		100	20		<0.05	Contig: Actin	GW7IPVU02F1G9T	*Placopecten magellanicus*	Mollusca, Bivalvia		MS-MS	
2123	42164		5.4		245	14		<0.05	Actin A3	gi|483321	*Aplysia californica*	Mollusca, Gastropoda		MS-MS	
	16066		6.6		164	28		<0.05	Contig: Actin	GW7IPVU02F1G9T	*Placopecten magellanicus*	Mollusca, Bivalvia		MS-MS	
2106	42002		5.3		154	52		7.7^e-10^	Actin 2	gi|18565104	*Crassostrea gigas*	Mollusca, Bivalvia		PMF	
	8125		9.9		21	18		<0.05	Contig: Actin	GW7IPVU02F2ZX3	*Crassostrea gigas*	Mollusca, Bivalvia		MS-MS	
2141 mixture	51678		5.2		105	40		6.1 ^e -5^	Retrograde protein of 51 kDa	gi|74912853	*Lymnaea stagnalis*	Mollusca, Gastropoda		PMF	
	41950		5.1		85	45		6.1^e-3^	Actin	gi|14010639	*Heliothis virescens*	Arthropoda, Insecta		PMF	
2150	42164		5.2		231	11		<0.05	Actin	gi|483321	*Aplysia californica*	Mollusca, Gastropoda		MS-MS	
	16066		6.6		140	20		<0.05	Contig: Actin	GW7IPVU02F1G9T	*Placopecten magellanicus*	Mollusca, Bivalvia		MS-MS	
2154	51678		5.2		141	37		1.5^e-8^	Retrograde protein of 51 kDa	gi|74912853	*Lymnaea stagnalis*	Mollusca, Gastropoda		PMF	
2158	42081		5.3		200	15		<0.05	Actin	gi|113290	*Aplysia californica*	Mollusca, Gastropoda		MS-MS	
	16405		4.6		82	11		<0.05	Contig: Actin	GW7IPVU02F1J4U	*Biomphalaria obstructa*	Mollusca, Gastropoda		MS-MS	
2174	42002		5.3		268	20		<0.05	Actin 2	gi|18565104	*Crassostrea gigas*	Mollusca, Bivalvia		MS-MS	
	16066		6.6		63	7		<0.05	Contig: Actin	GW7IPVU02F1G9T	*Placopecten magellanicus*	Mollusca, Bivalvia		MS-MS	
2218	42185		5.4		82	32		0.011	Actin	gi|47116422	*Biomphalaria pfeifferi*	Mollusca, Gastropoda		PMF	
2219	42002		5.3		75	49		0.05	Actin 2	gi|18565104	*Crassostrea gigas*	Mollusca, Bivalvia		PMF	
2220		42147		5,3	92	22		1.2^e-3^	Actin 5	gi|728796	*Limulus polyphemus*	Arthropoda, Merostomata	Cytoskeleton and muscle movements		PMF
2235		42002		5.3	182	67		1.2^e-12^	Actin 2	gi|18565104	*Crassostrea gigas*	Mollusca, Bivalvia			PMF
		14602		5.2	74	52		1.6^e-3^	Contig: Actin	GW7IPVU02HXDAX	*Nasonia vitripennis*	Mollusca, Gastropoda			PMF
2294		42310		5,16	84	25		7.8 ^e-3^	Skeletal muscle Actin 8	gi|207298839	*Homarus americanus*	Arthropoda, Malacostraca			PMF
2310		32663		4,58	193	48		<0.05	Tropomyosin-2	gi|1174755	*Biomphalaria glabrata*	Mollusca, Gastropoda			MS-MS
2393		32663		4,58	170	48		<0.05	Tropomyosin-2	gi|1174755	*Biomphalaria glabrata*	Mollusca, Gastropoda			MS-MS
2506		32663		4,58	119	11		<0.05	Tropomyosin-2	gi|1174755	*Biomphalaria glabrata*	Mollusca, Gastropoda			MS-MS
2844		49769		5,8	78	26		0.027	Similar to AGAP007334-PA, partial	gi|91095419	*Tribolium castaneum*	Arthropoda, Insecta			PMF
2927		49769		5.8	85	31		6.5^e-3^	Similar to AGAP007334-PA, partial	gi|91095419	*Tribolium castaneum*	Arthropoda, Insecta			PMF
3036		42081		5,3	91	40		1.6 ^e-3^	Actin	gi|113290	*Aplysia californica*	Mollusca, Gastropoda			PMF
514		79811		9.3	80	17		0.02	GF20923	gi|194763888	*Drosophila ananassae*	Arthropoda, Insecta	Post-transcriptional modifications		PMF
748		19214		5,37	91	54		1.6 ^e-3^	GA13613	gi|198472675	*Drosophila pseudoobscura*	Arthropoda, Insecta			PMF
1050		73710		4,8	146	5		<0.05	78 kDa glucose-regulated protein	gi|3023914	*Aplysia californica*	Mollusca, Gastropoda			MS-MS
1375		12501		8,27	78	61		0.034	cAMP-dependent protein kinase	gi|204305500	*Nasonia longicornis*	Mollusca, Gastropoda			PMF
1579		5046		5,5	68	13		6.1^e-3^	Contig: Galectin 4-like protein transcript variant	GW7IPVU02IO057	*Haliotis discus hannai*	Mollusca, Gastropoda			PMF
3769		42530		6,57	76	24		0.05	GE10781	gi|195504707	*Drosophila yakuba*	Arthropoda, Insecta			PMF
732		133246		8,66	87	14		3.8 ^e-3^	hypothetical protein TcasGA2_TC005757	gi|91088617	*Tribolium castaneum*	Arthropoda, Insecta	Chromosome segregation		PMF
1163		200851		6,34	78	17		0.031	GI21295	gi|195124758	*Drosophila mojavensis*	Arthropoda, Insecta			PMF
3529		80576		6,75	81	14		0.015	Similar to ATP-dependent DNA helicase PIF1	gi|221111106	*Hydra magnipapillata*	Cnidaria, Hydrozoa			PMF
		2435		5,4	73	32		2.2^e-3^	Contig: Unnamed	GW7IPVU02HXBVQ	*-*	-			PMF
4576		99375		9,47	83	20		9^e-3^	PIWI	gi|157116679	*Aedes aegypti*	Arthropoda, Insecta			PMF
861		2060		7	60		18	0.042	Contig: Cytochrome c oxidase subunit 1	GW7IPVU02G8789	*Albinaria caerulea*	Mollusca, Gastropoda	Oxidative phosphorylation		PMF
888		155613		6,34	82		21	0.014	Hypothetical protein CBG19638	gi|268563428	*Caenorhabditis briggsae*	Namatoda, Secernentea			PMF
		2060		7	60		18	0.041	Contig: Cytochrome c oxidase subunit 1	GW7IPVU02G8789	*Pulmonata sp.*	Mollusca, Gastropoda			PMF
4572		28835		9,05	80		44	0.021	WNT16	gi|284157247	*Capitella teleta*	Annelida, Polychaeta	Signalisation		PMF
2298		39305		7,12	125		6	<0.05	Arginine kinase	gi|3183057	*Liolophura japonica*	Mollusca, Polyplacophora	Energy storage		MS-MS
2089		15152		5,3	59		14	0.05	Contig: Alanine aminotransferase 2	GW7IPVU02IYGTU	*Crassostrea gigas*	Mollusca, Bivalvia	Detoxification		PMF
1627		108020		5,32	76		20	0.043	GF13819	gi|194758182	*Drosophila ananassae*	Arthropoda, Insecta	RNA Silencing		PMF
		2576		7,1	21		40	<0.05	Contig: Unnamed	GW7IPVU02FY4MI	*-*	-	-		MS-MS
3255		18856		4,6	47		5	<0.05	Ovipostatin	gi|283137716	*Lymnaea stagnalis*	Mollusca, Gastropoda	Egg Laying inhibition		MS-MS
3567		27606		5,42	45		10	<0.05	Yolk Ferritin	gi|1169743	*Lymnaea stagnalis*	Mollusca, Gastropoda	Egg Yolk protein		MS-MS
		12457		5,2	24		13	<0.05	Contig: Yolk Ferritin	GW7IPVU02F8ZI0	*Lymnaea stagnalis*	Mollusca, Gastropoda			MS-MS

Most of the spots identified were structural proteins mostly involved in the structure and function of the cytoskeleton (e.g., actin and tropomyosin) (Table 2). Expression of these proteins in the reproductive organs of *L. stagnalis* was downregulated by at least one of the tested chemical. Only one protein spot (i.e., spot n° 1411), corresponding to a retrograde protein of 51 kDa involved in intermediate filament constitution in *L. stagnalis*, was upregulated in individuals exposed to the highest concentration of chlordecone (19.6 µg/L) compared to solvent controls. At a lower chlordecone concentration (2.1 µg/L), 2 spots (i.e., spots n° 2141 and 2154) were downregulated and identified as retrograde protein of 51 kD. Among the structural protein identified as being altered following exposure to chemicals, 2 spot (i.e., spots n° 2129 and 2154) were downregulated following exposure to at least one concentration of each chemical tested (Table S1). Proteins involved in transcription and in post-transcriptional modifications of proteins, constitute a second major group that was downregulated after exposure to the chemicals. The molecules also affected proteins involved in signal transduction, cell division, energy storage, detoxification and oxidative phosphorylation (Table 2).

Finally, several proteins involved in reproduction showed significantly altered expressions in reproductive organs of *L. stagnalis* exposed to the endocrine disrupting chemicals tested. In reproductive organs of individuals exposed to 1.1 µg/L of cyproterone acetate, to 94.6 ng Sn/L of TBT and to 19.6 µg/L of chlordecone, the expression of PIWI, a key protein in germline stem cell differentiation [61], was significantly increased when compared to solvent controls (Table S1). An increase in the expression of another protein involved in *L. stagnalis* reproduction, ovipostatin (produced in the prostate gland [62]), was observed in individuals exposed to 19.6 µg/L of chlordecone compared to water controls (Table S1). However, this effect was not significant when compared to solvent controls. Lastly, yolk ferritin was downregulated compared to both controls following exposure to 94.6 ng Sn/L of TBT (Table S1). In *L. stagnalis*, yolk ferritin is the major egg yolk protein [63], suggesting that a reduction of expression of this protein could be responsible for a decrease in egg production or of the egg quality (i.e., reduction of energetic reserves for embryonic development). Therefore, to confirm the results obtained by 2D-DIGE, further analysis of the yolk ferritin expression was conducted using western blot, which is a more specific method for protein expression analysis.

### 3: Western Blot

#### 3.1. Antibody Efficiency

**Figure 3:**
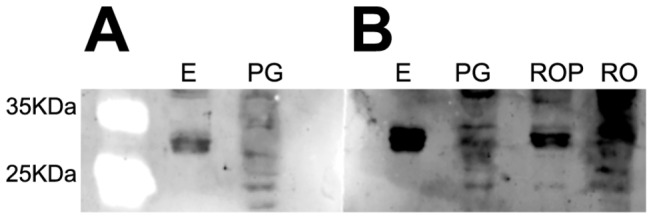
Western blot for yolk ferritin of 25 µg samples of proteins extracted from eggs (E), prostate gland (PG) and reproductive organs with (RO) and without prostate gland (ROP) incubated with (A) pre-immune rabbit serum and (B) post-immune rabbit serum.

Rabbit serum was used without purification of the anti-yolk ferritin antibody. The specificity of the yolk ferritin detection was confirmed by the identification of a band at the corresponding molecular weight (i.e., 27KDa) with further confirmation using positive and negative controls. Yolk ferritin is the main constituent protein of egg albumen in *L. stagnalis* [63,64]. Therefore, egg proteins were extracted and loaded on the gels as positive. The prostate gland is a male reproductive gland and probably does not synthetize nor stores this protein. Therefore, this gland was used as negative control on the gels. Results showed a strong expression of a band corresponding to a protein with a molecular weight between 25 and 35 KDa (Figure 3), that was observed in eggs but was weak or absent in the prostate samples. The pre-immune rabbit serum, sampled before the immunisation with the synthetic peptide, was tested on egg proteins and compared to the post-immune serum response. A weak detection of a band similar to the yolk ferritin protein was detected in egg samples after incubation with pre-immune serum, however the intensity of the band was much stronger in samples incubated with the post-immune serum (Figure 3). Finally, we have tested the serum on reproductive organs samples with or without the prostate gland. The removal of the prostate gland improved the detection of yolk ferritin (Figure 3). Therefore, in order to reduce unspecific binding, we have removed the prostate gland from the rest of the reproductive organs and negative controls (i.e., prostate gland proteins) were added to each gel. 

#### 3.2. Yolk Ferritin Expression in *Lymnaea stagnalis* Exposed to EDCs

**Figure 4:**
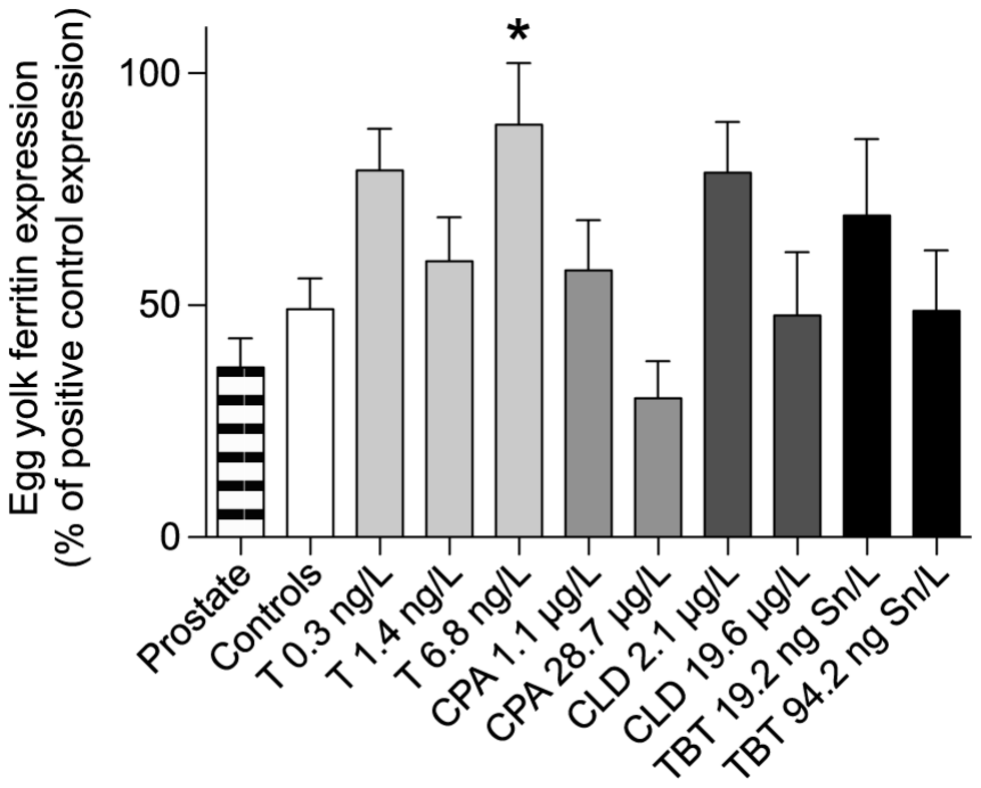
Yolk ferritin expression in reproductive organs of *L. stagnalis* exposed to testosterone (T), cyproterone acetate (CPA), chlordecone (CLD) and tributyltin (TBT) for 21 days. Results are expressed as percentage of expression measured in positive controls (egg samples) run with each gel. Error bars are SEM (*n*=4). Statistical differences from the controls; *: p<0.05.

Results of the yolk ferritin quantification were normalised between gels as percentages of the yolk ferritin expression signal detected in the positive control (i.e., proteins extracted from eggs sampled in our laboratory breeding stock), which was identical in every gel. Our results showed that, as previously observed during the method validation, a low but significant expression of yolk ferritin immunolabelling was found in the prostate gland (36.6 ± 6.3 % of the positive control yolk ferritin expression) (Figure 4). No statistical differences were observed between water and solvent controls (p= 0,092, Mann-Whitney test). Therefore water and solvent controls were pooled for subsequent statistical analysis as suggested in some guidelines for the assessment of the impacts of EDCs in molluscs [19]. The results obtained following exposure of *L. stagnalis* to EDCs were compared to the results obtained in the negative controls (i.e., proteins of a prostate gland from a snail from our laboratory breeding stock).

Only individuals exposed to 0.3 and 6.8 ng/L of testosterone and to a concentration of 2.1 µg/L of chlordecone showed a significantly higher percentage of yolk ferritin expression compared to the negative controls (i.e., prostate). Individuals exposed to the highest tested concentration of testosterone (6.8 ng/L) produced significantly more yolk ferritin than controls (p<0.05, ANOVA Dunnett’s *post hoc* test). Contrary to the downregulation in the yolk ferritin expression observed by 2D-DIGE analysis following exposure to 94.2 ng Sn/L of TBT, results of the western blot analysis showed that this treatment had no impacts on the protein expression.

## Discussion

### 1: Differential Proteomic Analysis

Our study is the first to investigate the impacts of putative endocrine disrupting chemicals on the proteome of the hermaphroditic freshwater gastropod *L. stagnalis*. This analysis was performed using the 2D-DIGE analysis, a method that allows assessment of the modification in expression levels of a large number of proteins with a good reproducibility [55]. Our results suggest that the four molecules tested, which can interact differently with the endocrine system of vertebrates, are able to alter the protein expression profile in reproductive organs of *L. stagnalis*. As compared to the solvent controls, the exposure of *L. stagnalis* to chlordecone had an impact on the expression of a greater number of proteins than the other compounds tested after 21 days of exposure. Moreover, the results of this study showed that proteins responsive to at least 2 chemicals or to different concentrations of a chemical were always altered in a same way (i.e., either up- or downregulation), suggesting that alteration in the expression of these proteins could be the result of a general stress response to chemical exposure [23]. Our results show that chemicals have impacts on different proteins suggesting that particular protein expression signature (PES) could be associated to each molecule as observed in other molluscs species exposed to different chemicals [24,29,65,66]. 

### 2: Impacts of EDCs on Reproductive Pathways

Many studies have reported that identification of specific protein expression signature (PES) is useful to propose new biomarkers of exposure to chemicals [29,65]. However, further identification of proteins disrupted by chemicals is essential to understand their mechanisms of action. The recent pyrosequencing of the genome of *L. stagnalis* [18] has helped to increase the number of proteins identified. Thus, seven more proteins were identified with this new genetic information additionally to proteins previously identified using other metazoa databases. Bouétard et al. [18] have reported that only 34.2 % of the contigs obtained by pyrosequencing *L. stagnalis* genome produced significant identifications using available databases. Therefore, some protein spots that significantly matched to the contigs could not be identified in this study. 

The majority of identified proteins were related to structure and function of the cytoskeleton (i.e., actin and tropomyosin). The high proportion of cytoskeleton proteins identified was also reported in other studies on mollusc species [67,68]. This is probably due to both their high abundance in molluscs databases and to their identification as major target of oxidative stress following exposure to pollutants in molluscs [23,68-70]. Even though *L. stagnalis* genome was sequenced, our results, along with the low proportion of contigs that produced significant identifications [18], support the need for gene annotation in gastropods to improve the results obtained from high throughput methods such as “omics” [71]. 

As previously observed on the proteome of different tissues of molluscs exposed to pollutants [23,68,70,72], actin and tropomyosin proteins were downregulated in the reproductive organs of *L. stagnalis* following a 21-day exposure to testosterone, cyproterone acetate, tributyltin and chlordecone. These proteins are highly abundant in muscle and non-muscle cells and impacts of toxicants on the expression of these proteins was suggested to be a consequence of the production of reactive oxygen species due to the toxicity of the compounds [68,70,73,74]. Therefore, the impacts of the tested chemicals on these proteins may not be attributed to endocrine disruption.

This study mainly focussed on the impacts of putative endocrine disruptors on the expression of proteins involved in mollusc reproduction. The 2D-DIGE analysis allowed the identification of protein expression alterations in the reproductive organs of *L. stagnalis*. An increase in PIWI expression was observed in reproductive organs of individuals exposed to cyproterone acetate (1.1 µg/L), tributyltin (94.6 ng Sn/) and chlordecone (19.6 µg/L) compared to solvent controls. PIWI is an evolutionary conserved protein, which is involved in the regulation in germ stem cell divisions [61,75,76]. It was observed in *Drosophila* *sp.* mutants that an overexpression of PIWI increased the division of germ stem cells, therefore increasing the number of gametes produced [61]. It was later reported that the role of PIWI was associated with spermatogenesis in vertebrates [77] as well as in invertebrates, such as the hermaphroditic *Caenorhabditis elegans* [78]. Upregulation in genes involved in spermatogenesis was observed in the gonochoric gastropod *N. lapillus* exposed to 100 and 200 ng Sn/L of TBT, using transcriptomic methods [71]. Therefore, the increase in PIWI expression observed in reproductive organs of *L. stagnalis* could indicate potential impacts of these chemicals on the gamete production, for example through alteration of spermatogenesis. In further studies, it would be interesting to investigate the impacts of cyproterone acetate, TBT and chlordecone on spermatogenesis along with the expression of PIWI in the reproductive organs of *L. stagnalis* exposed.

The expression of another protein, ovipostatin, was significantly increased in the reproductive organs of individuals exposed to 19.6 µg/L of chlordecone compared to water controls. This protein was recently isolated from seminal fluid of *L. stagnalis* and was reported to decrease oviposition when injected to the animals [62]. A recent study showed that ovipostatin also induces oligospermia in receptor individuals [79]. It is to be noted that at the end of the 21 days of exposure to chlordecone, a significant reduction of the number of clutches laid by snails exposed to 19.6 µg/L was observed [32]. These observations indicate that the reproductive impacts observed following exposure to chlordecone might be a consequence of an alteration of ovipostatin production by the prostate gland in *L. stagnalis*. Further analyses of the impacts of EDCs on *L. stagnalis* reproduction along with ovipostatin production by the prostate gland of *L. stagnalis* are required to confirm our results, as the change in abundance was not observed when compared to solvent controls. Furthermore, the mechanisms of action of ovipostatin on snail reproduction through endocrine pathways have to be further investigated. 

In *L. stagnalis* exposed to 94.2 ng Sn/L of tributyltin for 21 days, the yolk ferritin expression was downregulated in the 2D-DIGE experiment. This protein is the main yolk protein in oocytes in this species [63,80], whereas vitellogenin (Vtg), the precursor of egg yolk proteins in many species of vertebrates and invertebrates [81], has never been reported in freshwater gastropods. Alterations of Vtg production were reported in male fish affected by intersex in field and laboratory studies [42,82,83]. Nowadays, this protein is used in fish as biomarker of exposure to oestrogenic molecules in the environment [84,85] and is currently used to screen oestrogenicity of chemicals in the OECD test guideline Test No. 229: Fish Short Term Reproduction Assay [86]. Laboratory and field studies have reported modifications of Vtg expression in mollusc species exposed to oestrogenic compounds (e.g., 4-nonylphenol, 17ß-oestradiol) [87-90]. Moreover, in the scallop *Chlamys farreri*, transcriptomic analysis have reported that low concentrations of benzo(a)pyrene (0.4 and 2 µg/L) increased Vtg gene transcription whereas a higher concentration (10 µg/L) tended to reduce Vtg genes [91]. Vtg expression was proposed as biomarker of exposure to oestrogens in bivalves populations [92]. 

Therefore, alterations of yolk ferritin expression observed by 2D-DIGE analysis in our study suggest that EDCs could interact with oocyte production and egg quality in *L. stagnalis*. Western blot analysis was used to confirm the protein identification [93] and to provide more accurate quantitative results on yolk ferritin expression after exposure to chemicals. In this study we showed that further isolation of anti-yolk ferritin antibody from rabbit serum is needed in order to avoid the unspecific binding we observed. The western blot analysis has shown that yolk ferritin expression was modified in the reproductive organs of snails exposed to testosterone for 21 days. Therefore, our results suggest that in the hermaphrodite gastropod species *L. stagnalis*, a concentration of 6.8 ng/L of testosterone is able to induce alterations of the expression of the main egg yolk protein in the reproductive organs of individuals exposed for 21 days. This results support the hypotheses that this vertebrate sex steroid hormone might play a physiological role in mollusc reproduction [35,36]. Indeed laboratory experiments have shown that exposure of gonochoric molluscs to testosterone led to alterations of oocytes in female bivalves [37]. Even though the impacts of other chemicals on the expression of yolk ferritin were not statistically significant, further investigations of the alteration of this protein in *L. stagnalis* are needed.

Our results highlight that chemicals known for their endocrine disrupting properties in vertebrates can significantly alter proteins involved in reproduction of *L. stagnalis*. Some of the proteins identified are involved in different mechanisms such as gamete production (i.e., PIWI), oviposition (i.e., ovipostatin) or vitellogenesis (yolk ferritin). As oviposition was decreased [32] while ovipostatin expression was upregulated, chlordecone might be considered to act as an endocrine disruptor through alterations of the oviposition pathways. In the same treatment condition, an increase in PIWI expression was observed, which suggest an increase in gamete production. These results highlight that chemicals can act on reproduction through different mechanisms of action, especially in hermaphrodite species. Further analysis of the alterations of the expression of proteins identified in this study using more specific methods (e.g., western blot) are needed to better understand the impacts and the mechanisms of action of EDCs on the reproduction of *L. stagnalis*. These further investigations will also help to define whether these molecules act as endocrine disruptors in gastropods. 

## Conclusions

This study is the first to investigate the impacts of chemicals known for their endocrine disrupting properties in vertebrates on the proteome of reproductive organs of the hermaphroditic freshwater gastropod *L. stagnalis*. Our results show that the high throughput proteomic method 2D-DIGE allows the investigation of the impacts of chemicals, known for their endocrine disrupting properties in vertebrates, on the protein expression in the reproductive organs of the freshwater hermaphrodite gastropod *L. stagnalis*. This method allowed the identification of proteins involved in the reproduction that were differentially expressed following exposure to the chemicals, therefore suggesting possible endocrine disruption mechanisms. Among these proteins, yolk ferritin appeared to be a potential biomarker of exposure to different EDCs as its homolog, vitellogenin, in fish and other molluscs. *L. stagnalis* is proposed as candidate species for the development of an OECD guideline on reprotoxicity tests with molluscs. This guideline will cover level 4 and level 5 tests (i.e., *in vivo* assays on entire animals, that provide data on adverse effects on endocrine relevant endpoints), as defined in the revised version of the OECD Conceptual Framework for Endocrine Disruptors Testing and Assessment [94]. The analysis of yolk ferritin expression could be a complementary tool to screen the EDC potential of toxicants at the sub-individual level (i.e., level 3 tests: *in vivo* assays providing data about selected endocrine mechanisms/pathways). However, the analytical method for yolk ferritin needs to be improved and validated. Then, further analysis of the impacts of EDCs on this protein should be conducted in order to increase our knowledge concerning the alterations that endocrine disruptors might induce in this species. 

## Supporting Information

Table S1 **Raw data of protein spots with at least 1.5-fold expression change (p<0.05, student *t*-test) in reproductive organs of *Lymnaeastagnalis* after 21 days of exposure to chemicals compared to either Water controls (Versus Water controls) or Solvent controls (Versus Solvent controls).**
(XLSX)
